# Severe tricuspid regurgitation after a horse kick: a case report of a rare cause of acquired valvulopathy

**DOI:** 10.1093/ehjcr/ytae691

**Published:** 2024-12-24

**Authors:** Michaël Dorge, Remi Deleuse, Anne-Catherine Pouleur, Maria Chiara Badii

**Affiliations:** Department of Cardiology, Cliniques Universitaires Saint-Luc, Avenue Hippocrate, 10, Brussels 1200, Belgium; Department of Cardiology, Cliniques Universitaires Saint-Luc, Avenue Hippocrate, 10, Brussels 1200, Belgium; Department of Cardiology, Cliniques Universitaires Saint-Luc, Avenue Hippocrate, 10, Brussels 1200, Belgium; Department of Cardiology, Cliniques Universitaires Saint-Luc, Avenue Hippocrate, 10, Brussels 1200, Belgium

**Keywords:** Massive tricuspid regurgitation, Tricuspid surgical repair, Blunt chest trauma, Case report

## Abstract

**Background:**

Many rare complications are associated with blunt chest trauma and right ventricular contusion. Among these, post-traumatic severe tricuspid regurgitation is a relatively rare clinical entity. Furthermore, only a few cases reported in the literature are associated with trauma due to kicking by a horse.

**Case summary:**

We present the case of a 56-year-old woman who was diagnosed with early massive tricuspid regurgitation caused by traumatic rupture of the anterior papillary muscle, which was successfully treated by surgical tricuspid repair. The patient had no symptoms suggestive of valvular dysfunction, which was incidentally detected on routine transthoracic echocardiogram following a horse kick.

**Discussion:**

The most commonly cited mechanism is an anteroposterior compression of the chest, causing a sudden increase in right ventricular pressure during the end-diastolic phase. The mean interval to diagnosis of traumatic tricuspid regurgitation is usually long, leading to a progressive right ventricular remodelling and deterioration of right ventricular function. As a result, surgical repair of the valve is often not possible and an early tricuspid valve replacement is required.

**Learning points**

- Post-traumatic severe tricuspid regurgitation is a rare complication associated with blunt chest trauma and right ventricular contusion. The mean interval to diagnosis of traumatic tricuspid regurgitation is usually long, leading to a progressive right ventricular remodelling and deterioration of right ventricular function. Surgical repair of the valve is often not possible and an early tricuspid valve replacement is required.
- The case highlights the importance of an early diagnosis of traumatic tricuspid valve rupture (using cardiac imaging, electrocardiogram, clinical suspicion, and laboratory tests) and an early surgical treatment to increase the feasibility of tricuspid valve repair and to prevent progressive deterioration of right ventricular function.

## Introduction

Blunt trauma with right ventricular (RV) contusion occurs in 20% of patients with chest trauma.^1^ Many rare complications are associated with this situation such as chordae and papillary muscle rupture, coronary artery dissection or thrombosis, arrhythmias including commotio cordis,^2^ septal and free wall rupture,^3^ pericardial effusion, tamponade, or rupture with cardiac herniation.^4^

Among these, post-traumatic severe tricuspid regurgitation is a relatively rare and under-diagnosed clinical entity. Most cases reported in the literature are related to high-velocity motor vehicle accidents.

The tricuspid valve is the most commonly involved valve due to its anterior position,^5^ and the initial clinical presentation can range from asymptomatic to cardiogenic shock.

Although the optimal timing of surgical intervention in patients with post-traumatic tricuspid regurgitation remains controversial, early surgical intervention (as soon as possible after diagnosis) is highly recommended to preserve RV function and increase the feasibility of tricuspid valve repair.^6,7^

Fortunately, the use of biomarkers, clinical suspicion, and echocardiography has led to an increase in the earlier diagnosis of tricuspid regurgitation due to blunt chest trauma.^6^

In addition, echocardiography (including 3D imaging) allows rapid and non-invasive assessment of the morphology of the defect, including the mechanism and severity of the regurgitation, and assists in the planning of valve repair surgery.^8^

Very few cases reported in the literature have been associated with blunt chest trauma from a horse kick.^9^

## Summary figure

**Figure:**
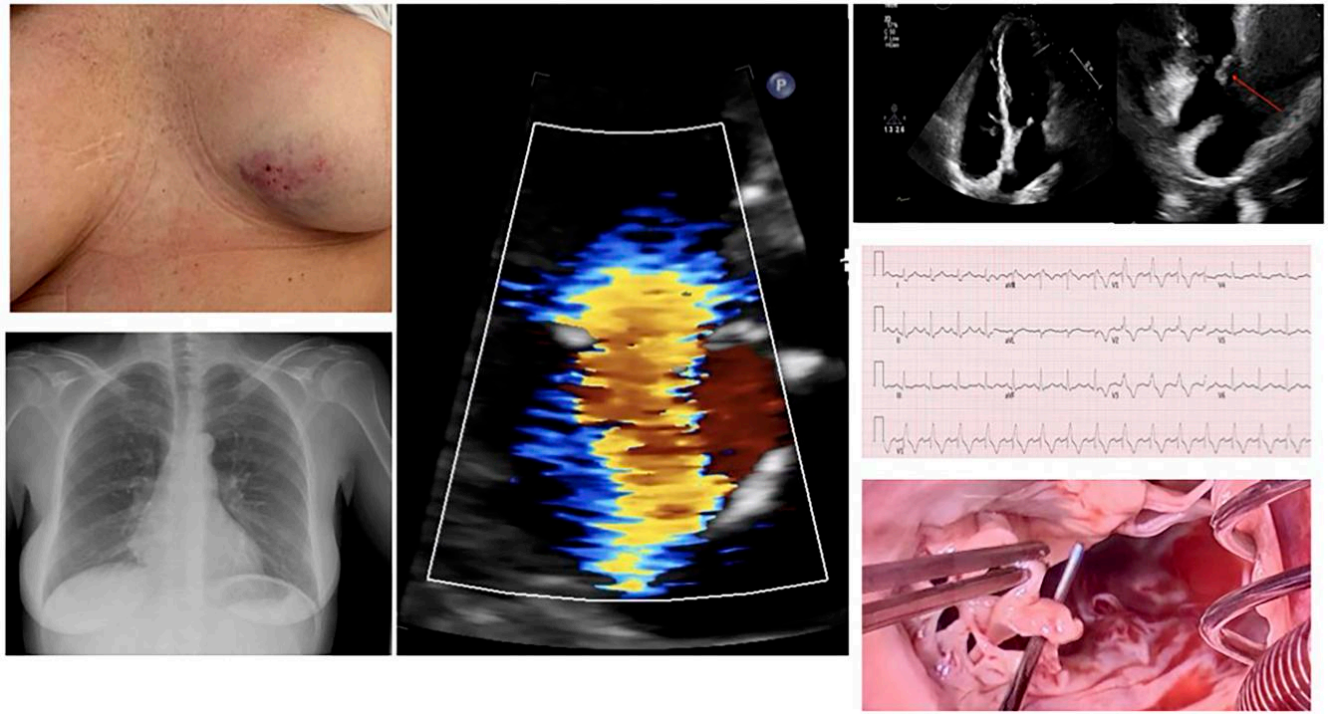


## Case summary

We report the case of a 56-year-old female patient who presented to the emergency department with vasovagal syncope due to severe pain following left parasternal thoracic trauma from a horse kick. Apart from the pain, the patient was completely asymptomatic.

**Figure 1:**
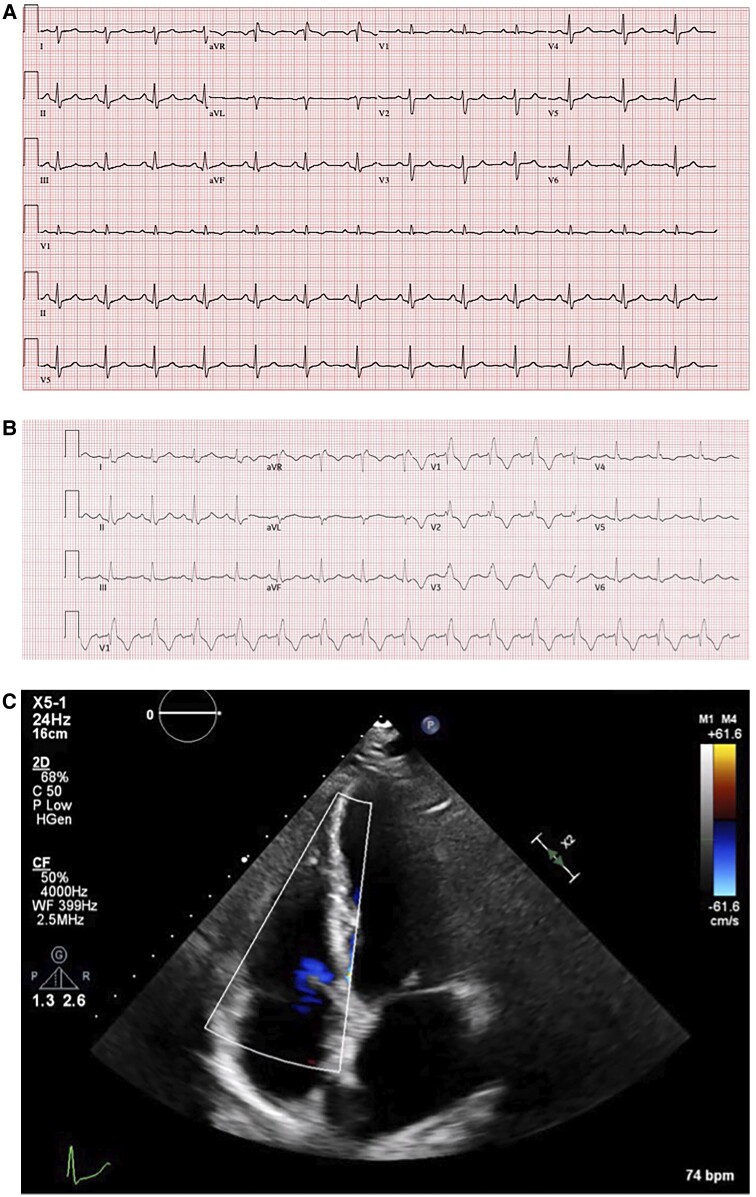
(*A*) Twelve-lead electrocardiogram prior the admission (2022). (*B*) Twelve-lead electrocardiogram at the admission in the emergency room showing a new complete right bundle branch block. (*C*) Transthoracic echocardiogram after surgical correction of atrial septal defect revealing a normal appearance of the tricuspid valve (2020).

Her pertinent medical history included surgical closure of a large ostium secundum atrial septal defect diagnosed at the age of 50. Her routine echocardiogram obtained before the horse kick showed normal RV dimensions without tricuspid valve abnormalities (*Figure 1C*; See Supplementary*Video S1*).

Long-term active smoking, estimated at 20 pack-years, was her only cardiovascular risk factor.

The initial clinical assessment in the emergency department was reassuring. Vital signs assessment revealed a heart rate of 90 b.p.m., blood pressure of 111/56 mmHg, and oxygen saturation (SpO2) of 100%. Physical examination revealed no murmurs and clear breath sounds without evidence of RV dysfunction. Chest radiography was normal without rib fractures or pneumothorax. A 12-lead electrocardiogram (ECG) showed sinus rhythm at 94 b.p.m. with a new complete right bundle branch block (QRS duration at 142 ms), which was absent on the previous ECG taken before the horse kick (*Figure 1A* and *B*).

Laboratory results showed a normal haemoglobin level of 13.8 g/dL (normal range 12.2–15.0 g/dL) and a normal creatinine level of 0.80 mg/dL (normal range 0.60–1.30 mg/dL). Troponin and N-terminal-pro-B-type natriuretic peptide levels were significantly elevated at 103 ng/L (normal range < 10 ng/L) and 838 pg/mL (normal range < 249 pg/mL), respectively.

No arrhythmias were observed during monitoring, and there was spontaneous regression of biomarkers of myocardial injury. Other monitored biological parameters (electrolytes, complete blood count, renal function, and basic coagulation tests) remained stable during the hospitalization.

**Figure 2:**
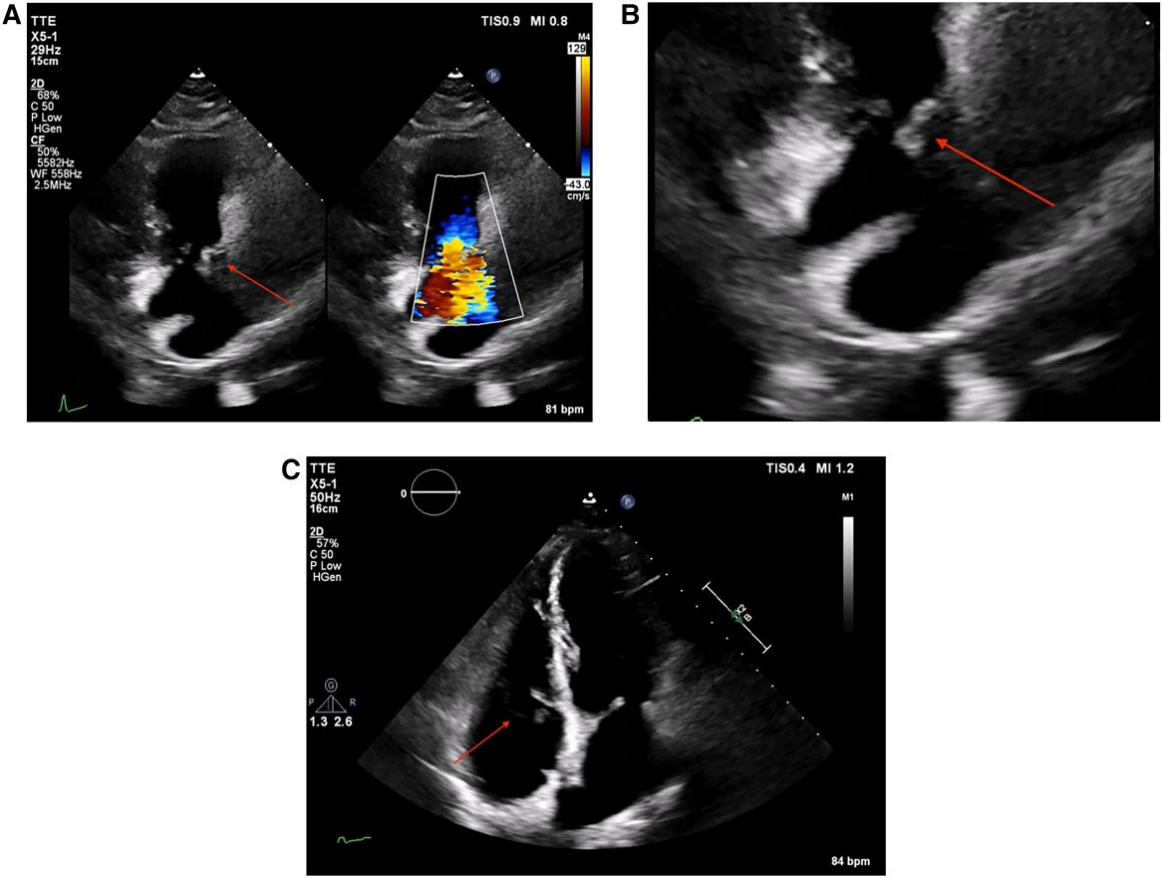
(*A–C*) Transthoracic echocardiogram showing severe tricuspid regurgitation due to the rupture of the anterior papillary muscle. Arrows indicating the flail leaflet.

A transthoracic echocardiogram (TTE) was performed the day after admission and showed massive (effective regurgitant orifice area 1.8 cm², vena contracta 14 mm), central, holosystolic tricuspid regurgitation due to the rupture of the anterior papillary muscle, with preserved RV function [fractional area change (FAC) 45.6%] and no other complications (*Figure 2*; See Supplementary*Videos S2–S5*). The diameter of the inferior vena cava was 20 mm, with inspiratory collapse of <50%.

**Figure 3:**
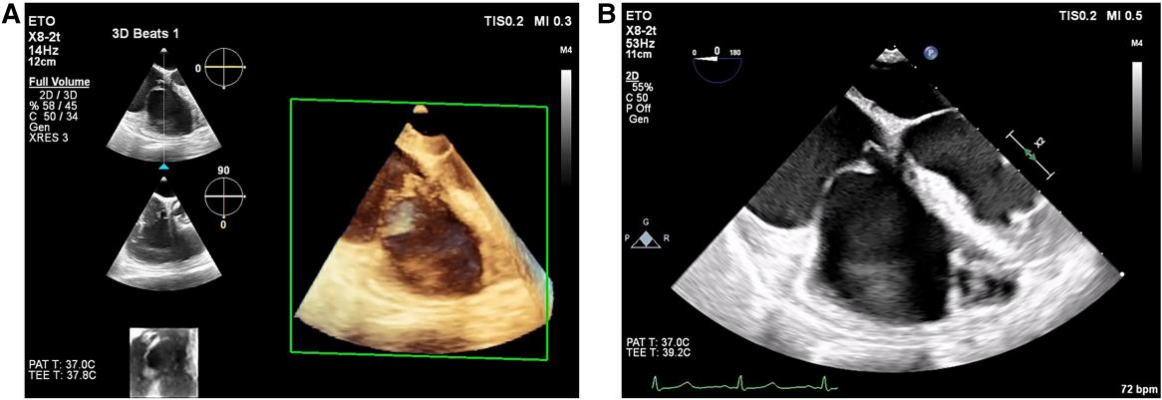
(*A*, *B*) Preoperative transoesophageal echocardiogram images.

**Figure 4:**
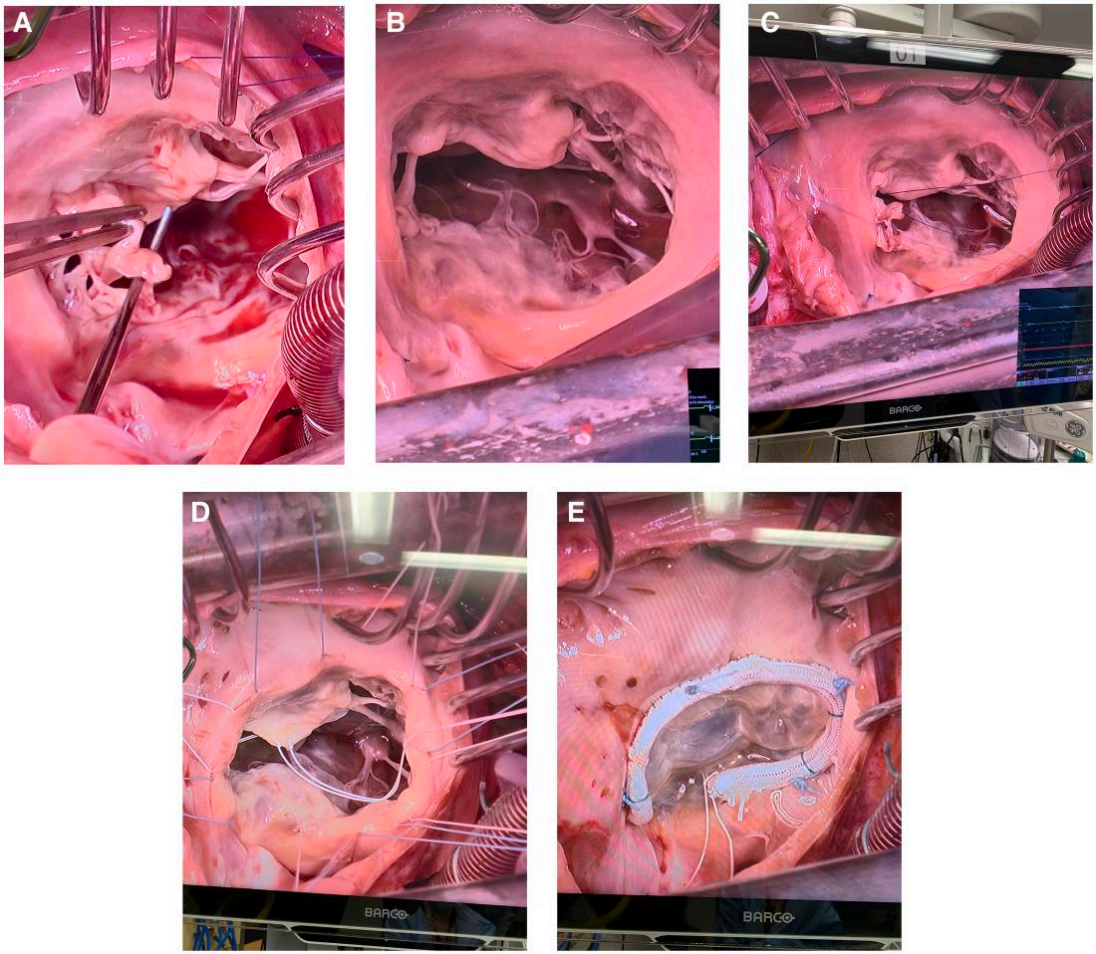
(*A–E*) Illustrations of the tricuspid valve repair surgery.

**Figure 5:**
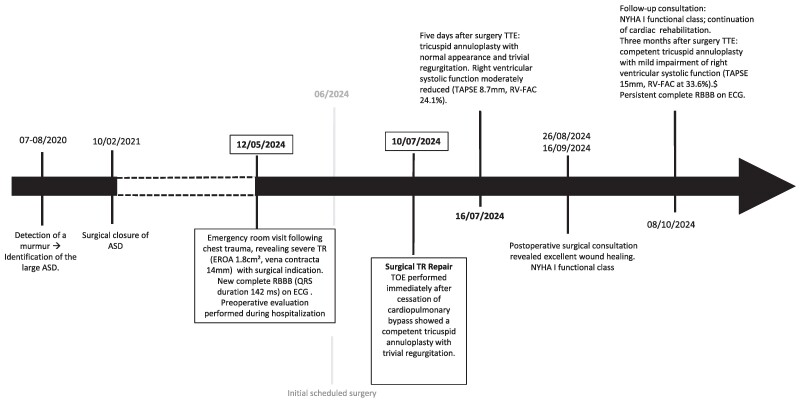
Key events timeline. ASD, atrial septal defect; ECG, electrocardiogram; EROA, effective regurgitant orifice area; NYHA, New York Heart Association; RBBB, right bundle branch block; RV-FAC, right ventricular fractional area change; TAPSE, tricuspid annular plane systolic excursion; TTE, transthoracic echocardiogram; TOE, transoesophageal echocardiogram; TR, tricuspid regurgitation.

After preoperative evaluation, she was scheduled for elective tricuspid valve repair (reimplantation of the anterior papillary muscle with neochordae and tricuspid annuloplasty) 3 weeks after the horse kick (*Figures 3* and *4*; See Supplementary*Videos S6–S8*). However, at the patient's request, the operation was postponed to 9 weeks after diagnosis (*Figure 5*).

The patient remained asymptomatic and without signs or symptoms of heart failure while awaiting surgery.

Transoesophageal echocardiogram (TOE) performed immediately after cessation of cardiopulmonary bypass showed a competent tricuspid annuloplasty with trivial regurgitation (See Supplementary*Video S9*).

Five days after surgery, the tricuspid repair was re-evaluated by TTE. The tricuspid annuloplasty had a normal appearance with trivial regurgitation, comparable with the post-bypass TOE findings. Right ventricular systolic function was moderately reduced [TAPSE (Tricuspid Annular Plane Systolic Excursion) 8.7 mm, RV-FAC 24.1%].

The TTE performed 3 months after surgery during the follow-up visit showed a competent tricuspid annuloplasty with mild impairment of RV systolic function (TAPSE 15 mm, RV-FAC at 33.6%).

At follow-up 3 months after surgery, the patient was in functional class New York Heart Association (NYHA) I and she was undergoing postoperative cardiac rehabilitation. Her ECG showed persistent complete right bundle branch block.

## Discussion

To our knowledge, traumatic rupture of the papillary muscle is a rare cause of tricuspid regurgitation and is mainly associated with high-speed motor vehicle accidents. After reviewing the literature, only a few cases related to horse kicks have been published. Therefore, this is a rare cause of traumatic tricuspid papillary muscle rupture.^9^

In contrast, several cases of mitral regurgitation following thoracic trauma have been reported.^5,10,11^

All of these cases are related to motor vehicle accidents with sudden deceleration.

As expected, the initial clinical presentation was often acute heart failure with haemodynamic instability, requiring urgent surgical intervention, with either mitral valve repair or replacement.

The most commonly cited mechanism of tricuspid regurgitation is an anteroposterior chest compression with a sudden increase in RV pressure during the end-diastolic phase, when the main pulmonary vessels are compressed. Significant traction is exerted on the valvular and subvalvular apparatus, leading to rupture of these structures.^12,13^

The mean time to diagnosis of traumatic tricuspid regurgitation is usually long, often several months or years, and the lesion is difficult to diagnose because of the slow progression of the disease.^9^

In addition, some publications report delayed tricuspid regurgitation due to papillary muscle contusion with haemorrhage, inflammation, and late necrosis, leading to dysfunction over time.^12,14^

Indeed, traumatic tricuspid valve rupture ranges from acute presentations to diagnoses made years after the blunt chest trauma,^13^ leading to a progressive RV remodelling and deterioration of RV function.

In this case, the diagnosis was made early, well before the onset of right heart failure symptoms.

In addition, the murmur of tricuspid regurgitation can be absent or inaudible, making diagnosis even more difficult.

In our case, transthoracic echocardiography performed within 24 h of the trauma played a key role in the diagnosis, in addition to the ECG changes (new onset of right bundle branch block), clinical suspicion, and biological findings.

The right bundle branch block is most commonly observed because of direct damage to the tricuspid valve and right heart muscle by blunt chest trauma,^5^ with or without left posterior fascicular block.^15^

Echocardiography (transthoracic or transoesophageal if necessary) should be performed in all patients with chest trauma, especially if cardiac injury is suspected, and in some cases even repeated if delayed tricuspid regurgitation is supposed.^12^

In all published cases, the final treatment was surgical. It is well established that repair is preferred to replacement due to better outcomes.^6,7^ However, when the tricuspid valve leaflets are tethered or the annulus is severely dilated, tricuspid valve repair can be challenging and not always possible, often leading to early tricuspid valve replacement.^7^

Thus, an earlier diagnosis followed by an earlier surgical treatment may increase the feasibility of tricuspid valve repair and prevent progressive deterioration of RV function,^15^ the development of atrial fibrillation, and other comorbidities that are associated with increased mortality.^7^

## Conclusion

We report the case of a patient who sustained blunt chest trauma from a horse kick, resulting in a traumatic rupture of the anterior tricuspid papillary muscle with severe tricuspid regurgitation. This complication was quickly suspected after the patient's admission due to elevated biomarkers of myocardial injury and ECG changes, although the patient was haemodynamically stable and quite asymptomatic.

This case illustrates a rare cause of severe acquired tricuspid regurgitation, in which an echocardiogram was performed within 24 h of the trauma, allowing early diagnosis and successful valve repair surgery before RV deterioration.

## Supplementary Material

ytae691_Supplementary_Data

## Data Availability

The data underlying this article will be shared on reasonable request to the corresponding author.

## Lead author biography

Dr Michaël Dorge graduated in 2018 from the Faculty of Medicine of the Université Catholique de Louvain, Belgium. He is currently a cardiology assistant at Cliniques Universitaires St Luc, Belgium. His main areas of interest are cardiovascular imaging and coronarography.

## Supplementary material

**Consent:** The authors confirm that written consent for the submission and publication of this case report was obtained from the patient in July 2024 according to the COPE guidelines.

**Funding:** None declared.
